# Recombinant human thrombopoietin as a novel platelet-driven regulator accelerating hepatic regeneration in acute liver failure

**DOI:** 10.3389/fphar.2025.1701928

**Published:** 2026-01-12

**Authors:** Yunzhi Shen, Fengzheng Han, Tao Wang, Li Jing, Ying Luo, Fushuang Ha, Yongping Lu, Jing Liang

**Affiliations:** 1 Department of hepatobiliary surgery, Tianjin Third Central Hospital, Central Hospital, Tianjin University, Tianjin, China; 2 Tianjin Key Laboratory of Extracorporeal Life Support for Critical Diseases, Institute of Hepatobiliary Disease, Tianjin University Central Hospital, Tianjin, China; 3 The Third Clinical College of Tianjin Medical University, Tianjin, China; 4 Department of Gastroenterology, Tianjin Binhai New Area Dagang hospital, Tianjin, China; 5 Department of hepatology and Gastroenterology, Tianjin Third Central Hospital, Central Hospital, Tianjin University, Tianjin, China; 6 Research Center for hepatobiliary diseases, Tianjin Third Central Hospital, Central Hospital, Tianjin University, Tianjin, China; 7 Department of Genetics, Liaoning Research Institute of Birth Health and Development, Shenyang, China; 8 Reproductive Hospital of China Medical University, Shenyang, China

**Keywords:** acute liver failure, platelet, recombinant human thrombopoietin, liver regeneration, rats

## Abstract

**Objective:**

The aim of this study is to investigate the effect of recombinant human thrombopoietin (rhTPO) on liver regeneration in rats with acute liver failure (ALF) induced by D-galactosamine (D-GalN).

**Methods:**

Sixty-six rats were divided into a control group and a TPO group. The control group received daily injections of normal saline, while the TPO group received daily injections of rhTPO. After five consecutive days of treatment, an ALF model was established in all rats via D-GalN administration. Survival status of the two groups was observed. Platelet count (PLT), liver function indicators, hepatocyte growth factor (HGF), and liver regeneration-related indicators were measured at different time points. Additionally, transcriptomic and proteomic analyses were performed on liver tissues.

**Results:**

Compared with the control group, the TPO group showed significantly higher levels of PLT, serum TPO, and HGF, milder liver tissue necrosis, a higher liver weight index, lower levels of alanine aminotransferase (ALT) and total bilirubin (TBil), and stronger liver regeneration capacity (as indicated by Ki67 and BrdU indices). Combined transcriptomic and proteomic analyses revealed that the expression of genes related to cell proliferation signaling pathways, such as Mapk1 and Map2k1, was significantly increased, while the expression of genes related to inflammatory pathways was significantly decreased.

**Conclusion:**

rhTPO can promote the recovery of liver function and enhance liver regeneration in ALF rats by increasing PLT, stimulating cell proliferation, and inhibiting inflammation.

## Graphical Abstract

**Figure:**
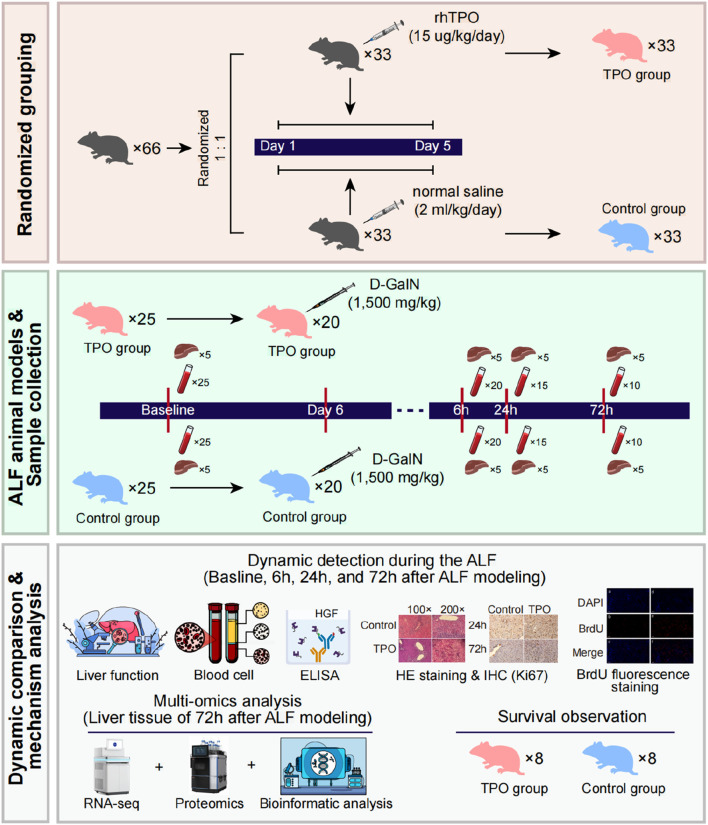

## 1 Introduction

Acute liver failure (ALF) is severe liver damage caused by various factors and manifests as severe liver dysfunction due to the rapid necrosis of many liver cells (Liver Failure and Artificial Liver Group, 2019). Currently, there are no cures for ALF, and the clinical mortality rate is 90% (Bernal and Wendon, 2013). Orthotopic liver transplantation is an effective method for the treatment of ALF. However, owing to the challenges associated with liver donation and the high cost of transplantation, the application of liver transplantation for ALF patients is severely limited (Zhang et al., 2016). Therefore, new treatments are urgently needed to improve ALF patient outcomes.

Liver regeneration plays a key role in the recovery of patients with liver failure (Wirth et al., 2018). An increasing number of studies have shown that, in addition to participating in hemostasis, platelets serve as important regulators of various physiological processes, such as immune regulation and tissue regeneration. Since platelets were first reported in 1982 to promote liver cell proliferation, a growing body of research has shown that platelets play an important role in liver regeneration after injury and hepatectomy (Lesurtel et al., 2006; Kirschbaum et al., 2015; Kirschbaum et al., 2017). Matsuo et al. (2011) demonstrated that platelet transfusion through the portal vein promoted liver regeneration in rats after 70% liver resection. In another study, microencapsulated platelet transfusion promoted liver regeneration in rats after 90% liver resection (Lopez et al., 2014). In addition, platelet transfusion can also increase the liver/body weight ratio and promote the proliferation of liver cells in rats after partial liver transplantation, accelerating liver regeneration after transplantation (Liang et al., 2021). Maruyama et al. (2013) demonstrated that platelet transfusion once a week for 12 weeks increased serum albumin (ALB), cholinesterase (CHE), and hyaluronic acid (HLA) levels in patients with chronic liver disease and liver cirrhosis, indicating that platelet transfusion may be beneficial for the treatment of liver fibrosis. However, platelet transfusion has several potentially severe side effects, such as infection, antiplatelet antibody production, anaphylaxis, and transfusion-related lung injury, which can all cause severe harm to patients (Pereboom et al., 2009).

Thrombopoietin (TPO) is the primary growth factor for platelet production, and the liver is an important site of TPO production. Recombinant human thrombopoietin (rhTPO) is structurally identical to endogenous TPO. It can specifically bind to the c-Mpl receptor and simultaneously activate three pathways—the JAK-STAT, MAPK, and PI3K–AKT pathways—to promote the proliferation, differentiation, and maturation of megakaryocytes, leading to the development of platelets (Nurden, 2011). However, there are no relevant reports on whether the application of rhTPO in patients with liver failure affects liver regeneration. Here, the effects of rhTPO on platelet production, recovery from liver injury, and liver regeneration were explored in an ALF model rat model.

## 2 Materials and methods

### 2.1 Experimental animal models and grouping

Sixty-six specific pathogen-free (SPF)-grade healthy male Sprague‒Dawley (SD) rats (6–8 weeks, body weight 190–210 g) were adaptively fed for 48 h in the animal laboratory and were randomly divided into a control group (33 rats) and a TPO group (33 rats). The control group was injected with normal saline (2 mL/kg/d) for five consecutive days. The TPO group was given a subcutaneous injection of rhTPO (15 μg/kg/d, Sansheng Pharmaceutical, Shenyang, China) for five consecutive days. On day 6, all rats were intraperitoneally injected with D-galactosamine (D-GalN, 1,500 mg/kg, Sigma, G1639) to establish the ALF rat model. After modeling, the mental status, appetite, activity, and defecation frequency of the rats were observed. Twenty rats (five rats per group) were sacrificed to collect blood and liver tissues at day 0 (baseline), 6 h, 24 h, and 72 h after modeling, respectively. Sixteen rats (eight in each group) were observed until 72 h after modeling, and the time to death and survival rate of the rats were recorded. All the rats were provided standard chow and water. The rats were housed at room temperature (20 °C–22 °C) under a 12 h/12 h light/dark cycle.

This study was reviewed by the Experimental Ethics Committee of Tianjin Third Central Hospital.

### 2.2 Experimental methods

To detect hepatocyte proliferation activity using the BrdU method, BrdU was administered to rats via intraperitoneal injection at a dose of 50 mg/kg body weight, 24 h before blood collection and liver tissue harvesting. Rats were anesthetized via inhalation of 5% isoflurane, and blood samples were collected through the orbital sinus puncture method. Half of the blood was treated with EDTA anticoagulation for complete blood cell analysis, while the remaining half was left at room temperature for 30 min and then centrifuged at 3000 *g* for 10 min to extract serum for biochemical index detection. Liver tissues were harvested immediately after blood collection, with all procedures strictly adhering to the guidelines for laboratory animal ethics to minimize animal discomfort and ensure scientific validity.

Albumin (ALB), total bilirubin (TBil), alanine aminotransferase (ALT), and aspartate aminotransferase (AST) levels were assessed via standard biochemical methods (Nanjing Jiancheng Bioengineering Institute, Nanjing, China). The serum hepatocyte growth factor (HGF) level was measured using the enzyme-linked immunosorbent assay (ERA21RB, Invitrogen, United States of America).

Immediately after blood collection, the livers were removed from the sacrificed rats, weighed, and incubated in 4% paraformaldehyde for approximately 24 h, followed by paraffin embedding. Liver tissue sections were prepared and stained with hematoxylin and eosin (HE). Pathological changes in the liver, including intralobular degeneration, focal necrosis, and inflammatory cell infiltration, were examined using an Olympus BX51 microscope attached to an Olympus DP71 digital camera and photographed. Pathological manifestations were semi-quantitatively graded according to each high-power field, range: 1+ (0–30%); 2+ (30%–60%); 3+ (>60%). The number of Ki67 (14–5698-82, Invitrogen, American)-positive cells in liver tissue was determined by immunohistochemistry. The Ki67 labeling index was calculated as the percentage (%) of Ki67-positive hepatocytes per 1000 hepatocytes. The number of BrdU (B23151, Invitrogen, United States of America)-positive cells in liver tissue was determined by immunofluorescence staining. The Ki67- and BrdU-positive cells were detected using Image Pro Plus software.

### 2.3 RNA sequencing and bioinformatic analysis

After 72 h of D-GalN induction, liver tissue was collected from each rat in the TPO group (n = 5) and the control group (n = 5) for mRNA sequencing to assess the differences in mRNA expression levels. mRNA sequencing was carried out by LC Bio Technologies (Hangzhou, China). In brief, total RNA was extracted using the TRIzol reagent (Thermo Fisher, 15596018), and the RNA integrity number was determined using a Bioanalyzer 2100 and the RNA 6000 Nano Kit (Agilent, CA, United States; 5067–1511). For the samples that met the quality standards, SuperScript II Reverse Transcriptase (Invitrogen, cat. 1896649, United States) was used for reverse transcription, yielding cDNA. The mRNA library was constructed using an NEB mRNA Stranded Library Preparation Kit (New England Biolabs, Beverly, MA, United States). In strict accordance with the manufacturer’s recommended protocol, 2 × 150 bp paired-end sequencing (PE150) was subsequently carried out on an Illumina NovaSeq 6000 platform by LC Bio Technologies (Hangzhou, China). After data quality control, the DESeq2 package in R was used to identify differentially expressed genes (DEGs), and then Gene Ontology (GO) and Kyoto Encyclopedia of Genes and Genomes (KEGG) enrichment analyses were performed on these DEGs.

### 2.4 Proteomic analysis

In parallel with mRNA sequencing, we also conducted a proteomic analysis of liver tissue from the TPO group (n = 5) and the control group (n = 5). Proteome analysis was performed by Scale Biomedicine Technology Co., Ltd. (Beijing, China). In brief, BSA standard protein solutions with gradient concentrations and sample solutions with different dilution factors were prepared. Bradford protein quantification and 12% SDS‒PAGE were performed. Next, the protein samples were treated with DB lysis buffer (8 M urea, 100 mM TEAB, pH 8.5), digested with trypsin, treated with formic acid, desalted using a C18 desalting column, and lyophilized. Then, an appropriate amount of peptide was taken from each sample and separated by chromatography using a Vanquish Neo UHPLC system (Thermo Scientific). The raw data were processed and analyzed using DIA-NN with the default settings.

### 2.5 Quantification of mRNA expression

Quantitative real-time PCR (qRT-PCR) was performed to validate the mRNA expression levels of target genes. The TB Green Premix Ex Taq II Kit (TaKaRa, China, Catalog No.: RR820A) was used, and all operations were strictly performed in accordance with the manufacturer’s instructions to ensure reagent stability and reaction specificity. The qRT-PCR assays were conducted on an 7500 Fast Real-Time PCR System (Applied Biosystems, American) using gene-specific primers. All primers used in this study were synthesized by Sangon Biotech (Shanghai, China). Glyceraldehyde-3-phosphate dehydrogenase (GAPDH) was used as the internal reference gene. The relative expression levels of target genes were calculated using the 2^-ΔΔCt^ method.

### 2.6 Statistical methods

SPSS 26.0 software was used for statistical analysis. Count data are expressed as the means ± standard deviations (SDs), and categorical variables are expressed as percentages (%). The chi-square test was used to compare qualitative data, and the Mann‒Whitney U-test was used to compare rank data. All statistics were two-sided, and *p* < 0.05 was considered to indicate statistical significance. The VennDiagram package in R software was used to identify the intersecting genes between the DEGs identified via mRNA sequencing, and those encoding differentially expressed proteins were identified via proteomic analysis.

## 3 Results

### 3.1 General observations of the rats

After modeling, 16 rats in the two groups exhibited low activity, poor mental status, and yellow urine, while some rats also showed orbital hemorrhage, loss of appetite, and reduced defecation. Two rats in the control group died within 48 h after modeling, and two rats in the TPO group died within 24 h after modeling. The surviving rats in the TPO and control groups showed improvements in mental status and activity at 3 days and 5 days after modeling, respectively. The level of activity almost returned to normal, and appetite also increased. The 72-h survival rate of the rats in both groups was 75% (2/8).

### 3.2 Changes in hemocyte levels in the control and TPO groups before and after modeling

**FIGURE 1:**
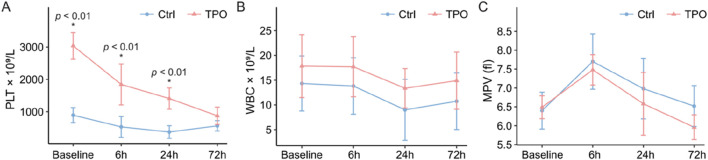
Comparison of blood parameters before modeling and at 6 h, 24 h, and 72 h after modeling. PLT **(A)**, WBC **(B)**, and MPV **(C)** levels in the control and TPO groups before modeling vs. 6, 24, and 72 h after modeling.

Routine blood tests were performed, and serum TPO levels were measured on day 6 before modeling. The platelet count (PLT) (3038.1 ± 412.6 vs. 893.6 ± 230.8, *p* = 0.000) and serum TPO level (552.9 ± 655.7 vs. 59.6 ± 30.4, *p* = 0.000) in the TPO group were significantly greater than those in the control group. The PLT was greater in the TPO group than in the control group at 6 h and 24 h after modeling (Figure 1A). There was no significant difference in the white blood cell count (WBC) or mean platelet volume (MPV) between the two groups (Figures 1B,C).

### 3.3 Liver weights of ALF model rats in the control and TPO groups

**FIGURE 2:**
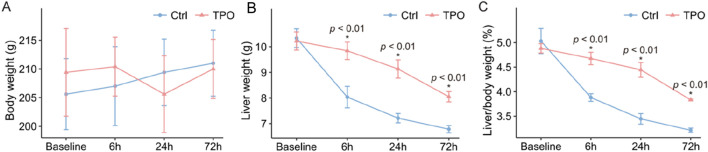
Liver weight index in ALF model rats following TPO treatment. Body weight **(A)**, liver weight **(B)**, and liver weight indices **(C)** of the rats in the TPO and control groups at 6 h, 24 h, and 72 h after modeling. Data are presented as the mean ± SEM; ns, not significant versus Ctrl (n = 5 per group).

The liver weight/body weight ratio, also known as the liver weight index (Zhang et al., 2016), was calculated for each group at all tested time points. There was no difference in liver weight indices between the two groups of rats that were not subjected to D-GalN treatment. Before modeling, the liver weight indices of the rats in the two groups were 5.03% and 4.88%, respectively. The body weight of ALF model rats in both groups showed no significant change after modeling (Figure 2A). The liver weights of ALF model rats in both groups tended to decrease after modeling (Figure 2B). The mean liver weight indices in the TPO group were significantly greater than those in the control group (4.7% vs. 3.9%, *p* = 0.001; 4.4% vs. 3.4%, *p* = 0.001; 3.8% vs. 3.2%, *p* = 0.001) at 6 h, 24 h, and 72 h after modeling, and the decreasing trend in the liver weight index after ALF modeling was more pronounced in the control group than in the TPO group (Figure 2C).

### 3.4 Comparison of the levels of liver function markers in ALF model rats between the control and TPO groups

**FIGURE 3:**
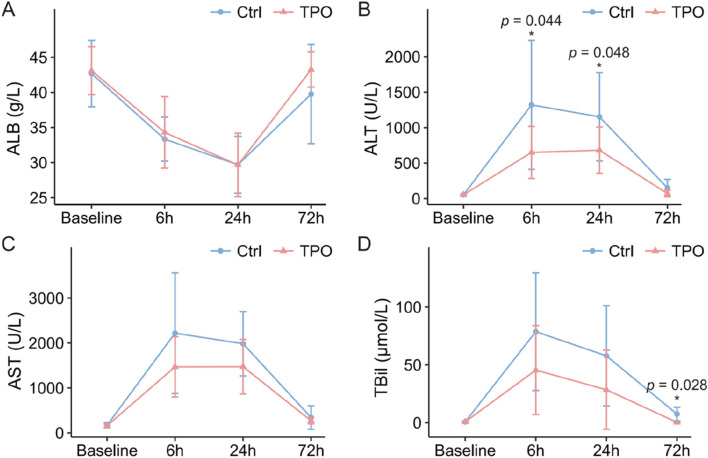
Serum liver-function markers in ALF rats after TPO treatment. ALB **(A)**, ALT **(B)**, AST **(C)**, and TBil **(D)** were measured at baseline and 6 h, 24 h, and 72 h after D-GalN administration. Data are presented as the mean ± SEM; ns, not significant versus Ctrl (n = 5 per group).

In the two groups, the ALT, AST, and TBil levels were significantly increased at 6 h after modeling (Figures 3B–D), whereas the ALB levels were significantly decreased at 24 h after modeling (Figure 3A). The ALT level in the TPO group was significantly lower than that in the control group at 6 h and 24 h (650.8 U/L vs. 1321.2 U/L, *p* = 0.044; and 682.0 U/L vs. 1152.7 U/L, *p* = 0.048) (Figure 3B). The TBil levels were significantly lower in the TPO group than in the control group (0.3 μmol/L vs. 7.4 μmol/L, *p* = 0.028) 72 h after modeling (Figure 3D), and there was no significant difference in levels of other indicators between the two groups at 72 h after modeling.

### 3.5 Comparison of serum HGF levels between the control and TPO groups

**FIGURE 4:**
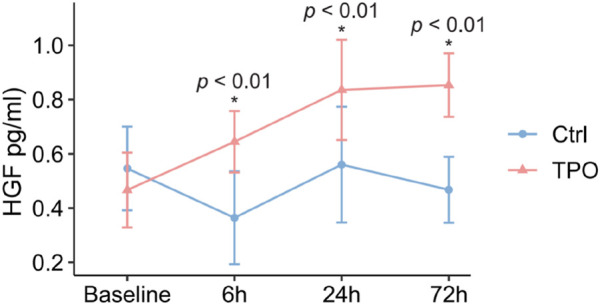
TPO elevates serum HGF in ALF rats. Serum HGF was quantified using ELISA before (baseline) and at 6 h, 24 h, and 72 h after D-GalN injection. Data are presented as the mean ± SEM; n = 6–8 per group.

Differences in serum HGF levels between the control and TPO groups, before modeling and at 6 h, 24 h, and 72 h after modeling, were measured using ELISA. The results revealed that HGF levels in the control group tended to decrease at 24 h after modeling, whereas HGF levels in the TPO group gradually increased after modeling; serum HGF levels at 6 h, 24 h, and 72 h after modeling were significantly greater than those in the control group (*p* < 0.01) (Figure 4).

### 3.6 Pathological changes in the liver tissues of ALF model rats in the control and TPO groups

**FIGURE 5:**
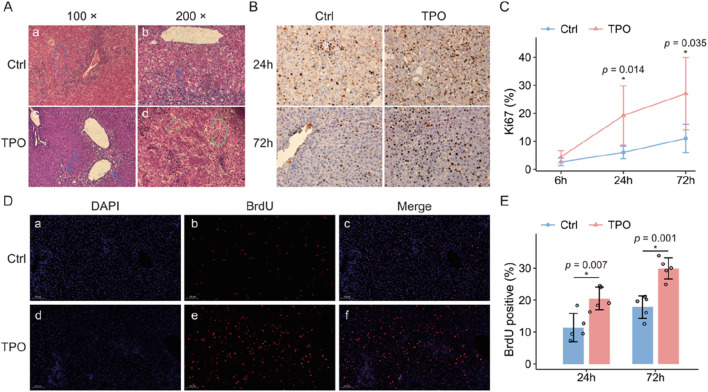
Pathological changes in the liver tissues of ALF model rats in the control and TPO groups at 24 h. **(A)** Pathological manifestations of liver tissues at 24 h in ALF model rats in the control and TPO groups. In the liver tissues of both the control and TPO groups of rats, hepatocyte swelling, necrosis, hemorrhage, and sinus dilation were observed. The black arrows indicate fatty degeneration, the blue arrows indicate hepatocyte necrosis and hemorrhage, and the green circles indicate sinus dilation. **(B)** Ki67 immunohistochemistry results of the control and TPO groups (400×). **(C)** Ki67 indices of the control and TPO groups at 6 h, 24 h, and 72 h after modeling. **(D)** BrdU fluorescence staining in the control and TPO groups at 72 h after modeling (red indicates BrdU-positive liver cell nuclear staining, 400x). Panels a, b, and c show the DAPI staining images, BrdU staining images, and merged images of liver tissue from rats in the control group, respectively. Panels d, e, and f show the DAPI staining, BrdU staining, and merged images of liver tissue from rats in the TPO group, respectively. **(E)** Percentages of BrdU-positive cells in the control and TPO groups.

ALF model rats in the two groups were sacrificed at 6 h, 24 h, and 72 h after modeling. Pathological changes in the liver tissues of the rats in both groups were observed. Lamellar necrosis and swelling of hepatocytes, collapse of the reticular scaffold, and hemorrhage in the hepatic sinusoids were observed at 6 h after modeling in both groups of ALF model rats. At 24 h, histopathological staining revealed obvious hepatocellular necrosis, liver cell swelling, diffuse steatosis, hepatic sinus dilatation, hemorrhage, and mesh stent collapse. Pathological examination of liver tissues from the two groups at 72 h revealed focal and lamellar necrosis, apoptosis, and inflammatory cell infiltration, which were alleviated compared with those at 6 h and 24 h. Liver tissue necrosis was milder in ALF model rats in the TPO groups than in those in the control group at 6 h and 24 h. However, there was no statistically significant difference between the semi-quantitative histopathological scores of the two groups of liver tissues. The pathological changes in the liver tissues of the rats in the two groups are shown in Figure 5A.

### 3.7 Comparison of the Ki67 proliferation indices between the control and TPO groups

Hepatocyte regeneration was evaluated via immunohistochemical staining of Ki67 in liver tissue with a recombinant anti-Ki67 antibody, DAB staining solution, and Harry’s hematoxylin staining solution (Figure 5B). The results revealed that there was no significant difference in the Ki67 proliferation index between the two groups at 6 h after modeling. However, over time, the Ki67 labeling indices at 24 h and 72 h after modeling in the TPO group were significantly greater than those in the control group (19.0% vs. 6.0%, *p* = 0.014; 27.0% vs. 11.0%, *p* = 0.035) (Figures 5B,C).

### 3.8 Comparison of the BrdU fluorescence detection results between the control and TPO groups

To assess the proliferation status of liver cells, BrdU fluorescence staining (BrdU monoclonal antibody, PBS phosphate-buffered saline, and DAPI nuclear stain solution) was performed on liver tissue sections from the two groups after 24 h and 72 h of modeling. Nuclei were positively stained in both groups, suggesting the presence of liver cells undergoing mitosis. Compared with those in the control group, the percentages of BrdU-positive liver cells in the TPO group were significantly greater at 24 h and 72 h (11.4 vs. 20.5, *p* = 0.007; 17.8 vs. 29.9, *p* = 0.001), as shown in Figures 5D,E.

### 3.9 Differential mRNA and protein expression profiles between the TPO and control groups

**FIGURE 6:**
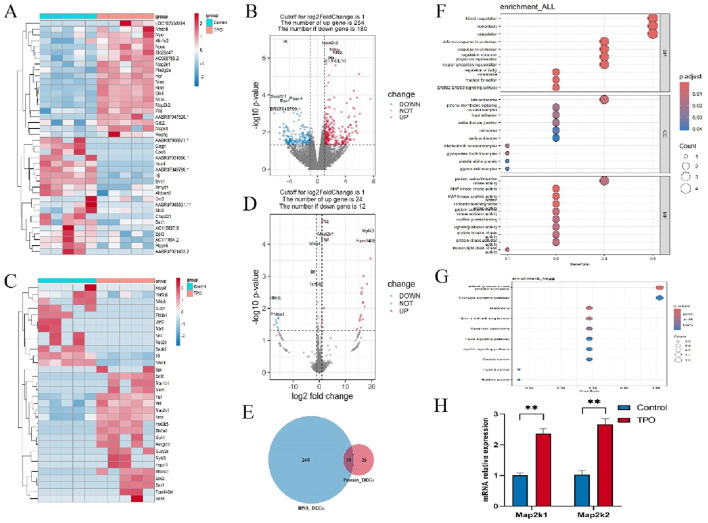
Results of mRNA sequencing and proteomic analyses. **(A,B)** Heatmap and volcano map of mRNA sequencing. **(C,D)** Heatmap and volcano map of proteomic analyses. **(E)** Venn diagram of the DEGs from transcriptomic and proteomic analyses. **(F)** GO enrichment analyses of the overlapping genes. **(G)** KEGG enrichment analyses of the overlapping genes. **(H)** mRNA expression of Map2k1 and Map2k2.

In this study, mRNA sequencing of liver tissues 72 h after D-GalN induction revealed that, compared with the control group, the TPO pretreatment group presented 262 upregulated genes and 188 downregulated genes (Figures 6A,B). Specifically, in the TPO treatment group, the expression levels of genes related to cell proliferation (such as *Mapk1* and *Map2k1*) and the hepatocyte growth factor (*Hgf*) gene were significantly increased, whereas the expression of the inflammatory factor (*IL-6*) was significantly decreased. These findings suggest that TPO may play a role in liver recovery by promoting hepatocyte proliferation and suppressing the inflammatory response.

Proteomic analyses of five pairs of liver tissues revealed 24 upregulated proteins and 12 downregulated proteins in the TPO pretreatment group (Figures 6C,D). Intriguingly, the proteomic data revealed that the protein levels of *Map2K1* and *Hgf* were significantly increased, whereas the level of *IL-6* was significantly decreased in the TPO pretreatment group. This outcome was consistent with the results of the transcriptomic analysis.

A Venn diagram analysis revealed that there were 10 overlapping genes between the DEGs from the transcriptomic and proteomic analyses (Figure 6E). GO and KEGG enrichment analyses of these 10 genes revealed that TPO may exert its effects through the MAPK signaling pathway (Figure 6F) and the PI3K‒AKT pathway (Figure 6G). To verify the reliability of our transcriptomic data, qRT-PCR was performed to detect the mRNA expression levels of Map2k1 and Map2k2. he results showed that the mRNA expression levels of Map2k1 and Map2k2 in the rhTPO-treated group were significantly higher than those in the control group (*p* < 0.05) (Figure 6H).

## 4 Discussion

A growing body of evidence from rodent and *in vitro* studies indicates that the function of platelets extends far beyond hemostasis; they also have immune regulatory functions and actively participate in liver inflammation (Nurden, 2011; Ripoche, 2011). Platelets enter the injured liver and interact with liver sinusoidal endothelial cells, which can induce the release of up to 300 biologically active proteins (including cytokines, chemokines, growth factors, and hemostatic proteins) from platelet alpha-granules (Morrell et al., 2014), along with important bioactive lipids. Through the secretion and release of a variety of bioactive molecules, platelets can participate in important physiological processes in the liver, ranging from the regulation of inflammation and fibrosis to liver repair and regeneration (Gruber et al., 2005; Gerard et al., 2007). Over the past 10 years, many studies have shown that platelets play important roles not only in thrombus formation and wound repair but also in liver regeneration, immune regulation, and cell proliferation. Multiple studies have shown that a low PLT is associated with delayed recovery of liver function after partial hepatectomy and liver transplantation (Bleibel et al., 2013). Commonly used methods to increase the PLT in clinical practice include splenectomy, embolization, and platelet transfusion. Due to limitations in the timing and storage of platelet collection, there is currently a lack of conclusive evidence on the true effect of platelet transfusion on promoting liver regeneration in humans. However, previous studies have confirmed the effect of splenectomy on liver regeneration in an experimental model of partial hepatectomy. Splenectomy can increase the PLT and increase HGF levels in rats, thereby accelerating liver regeneration after partial hepatectomy (Tomikawa et al., 1996; Lee et al., 2015).

Previous studies have focused mainly on PLT and liver regeneration after hepatectomy and liver transplantation. Some early studies also indicate the inhibitory effect of platelets on liver fibrosis in a liver cirrhosis rat model (Markham, 2021; Murata et al., 2008). In patients with liver cirrhosis, platelet transfusion can also improve liver function (Maruyama et al., 2013). However, studies investigating whether platelets play a role in liver regeneration in patients with liver failure have rarely been reported.

ALF is a syndrome characterized by severe liver dysfunction and high mortality. The interaction between liver cell necrosis and regeneration often occurs during the process of liver failure. Effective liver regeneration plays a key role in the prognosis of liver failure. With respect to the protective effect of platelets after hepatectomy, we hypothesized that treatments that increase the PLT may facilitate liver function recovery and liver regeneration in rats with ALF. Currently, TPO and TPO receptor agonist injection is considered a feasible alternative to platelet transfusion for increasing the PLT. We used rhTPO as a platelet production promoter. Our preliminary experiments revealed that TPO effectively increased the PLT. In addition, our research group reported the effect of rhTPO on increasing the PLT in patients with liver cirrhosis (Liang et al., 2016). Preliminary experimental results revealed that the PLT in rats with liver failure significantly increased to a peak value 5 days after TPO administration. Therefore, based on the previous experimental results, we injected rhTPO 5 days before modeling to increase the PLT. The effects of an elevated PLT on liver function and liver regeneration in ALF model rats have been previously observed (Tang et al., 2024).

In the current study, there were significant differences in the PLT between the two groups of rats before modeling. The rats in both groups presented different degrees of notable liver injury 6 h after modeling. The early pathological changes in the liver tissues of the rats in the two groups were extensive necrosis and inflammation of liver cells, including dilatation of the liver sinus, hemorrhage, and inflammatory cell infiltration. After 72 h, liver cell necrosis was slightly ameliorated, and the degree of liver injury in the TPO group was milder than that in the control group. Previous studies have only included histological assessments based on the pathological descriptions of the liver and lack quantitative evaluation indicators. To this end, we compared the liver weights of two groups of ALF model rats and showed that liver weight reduction can occur in ALF and is related to the degree of necrotic injury to liver tissue. We observed that the liver weights gradually decreased during ALF in the two groups of rats, which was also one of the reasons for the decrease in TPO levels. However, the liver weights of the rats in the TPO group at 6 h, 24 h, and 72 h were significantly greater than those of the control group. In animal experiments involving hepatectomy and liver transplantation injury, the increase in liver weight after TPO injection was related to the protective effect of platelets on liver injury and liver regeneration (Liang et al., 2016; Tang et al., 2024). Moreover, we compared the results on the levels of liver function markers between the two groups. These findings suggested that the increases in AST and TBil levels at 24 h were the most pronounced in ALF model rats. Compared with the control group, the ALF model rats in the TPO group exhibited less severe liver injury and faster recovery of liver function.

In ALF, liver cell death usually induces compensatory cell proliferation to increase liver weight and prevent the loss of organ function (Liang et al., 2021). The balance between liver regeneration and liver cell death is a key factor affecting the prognosis of ALF (Lesurtel et al., 2006)^.^ Therefore, in addition to comparing necrosis and inflammation in the liver, we also observed indicators of liver cell regeneration and proliferation in liver tissue samples. Previous studies have included assessments of hepatocyte proliferation by Ki67 and BrdU staining (Kirschbaum et al., 2015; Kirschbaum et al., 2017; Shimabukuro et al., 2009). In this study, compared to control rats, D-GalN-induced ALF model rats had a significantly greater percentage of both Ki67-and BrdU-positive cells in the liver after rhTPO injection, suggesting that the proportion of proliferating liver cells was increased in the TPO group. The results of this study were consistent with those of recent studies in rats subjected to liver transplantation. This effect was subsequently attenuated by treatment with anti-platelet drugs to reduce the PLT; notably, it has been proposed that platelets have an important promoting effect on liver cell proliferation and regeneration (Liang et al., 2021). A latest study also found that in acetaminophen (APAP)-induced liver injury in mice, the novel thrombopoietin mimetic peptide can arrest the progression of liver injury and accelerate the hepatocyte proliferative response (Adelusi et al., 2024).

In this study, serum HGF levels in the rats of the TPO group were significantly increased at 24 h after modeling, and the levels at 24 and 72 h were significantly greater than those in the control group, which was consistent with the Ki67 and BrdU staining trends, suggesting that the increase in the PLT induced by TPO may promote the release of HGF and facilitate liver regeneration.

We also performed transcriptome sequencing and proteomics analysis of liver tissues from ALF mice treated with TPO. The results revealed that the changes in the expression of numerous genes in TPO-treated mice were consistent at both the mRNA and protein levels. This concordant expression variation not only highlights the robustness of the multiomics approach but may also reveal key molecular factors critical to the biological phenomena under investigation; these findings provide a foundation for further research on the functional implications and regulatory roles of these potential key factors. Both the mRNA and protein expression levels of HGF significantly increased, which was consistent with the elevated HGF levels in the serum, further confirming that TPO can promote the expression and release of HGF. Additionally, genes related to cell proliferation signaling pathways (Mapk1 and Map2k1) were also significantly upregulated in TPO-pretreated mice, indicating that TPO may also promote liver regeneration by promoting hepatocyte proliferation. The qRT-PCR results confirming elevated Map2k1 and Map2k2 expression in the rhTPO-treated group are highly consistent with our transcriptome sequencing and proteomics analysis. These consistent results further support that rhTPO regulates the MAPK pathway by modulating these key genes. Multiple studies have shown that activation of the MAPK signaling pathway can alleviate symptoms of acute liver failure (Roy et al., 2017; Najimi et al., 2009). Additionally, we found that the expression levels of inflammatory factors such as IL-6 were decreased at both the mRNA and protein levels, suggesting that, in addition to promoting cell proliferation, TPO may also alleviate liver damage by inhibiting liver inflammatory responses.

In summary, liver regeneration associated with TPO-induced platelet elevation was investigated in ALF model rats. Platelets can increase the level of HGF, promote the recovery of liver function and cell proliferation, and contribute to liver regeneration. Based on these findings, it is expected that, in clinical practice, treatments aimed at increasing the PLT will provide new options for the treatment of liver failure.

Although the important role of platelets in liver regeneration has been confirmed in many animal experiments, studies on the role of platelets in improving liver function and promoting liver regeneration are still in their infancy. Although there is evidence that an increased PLT facilitates liver function recovery and liver regeneration after hepatectomy and liver transplantation, evidence for their role in liver failure is still lacking. In this study, D-GalN was used to establish an ALF model rat model; the study revealed that TPO injection promoted liver function and liver regeneration in ALF model rats. However, further in-depth studies are needed to explore the specific mechanism involved.

## Data Availability

The raw mRNA sequencing data can be found in the NCBI BioProject database, accession number PRJNA1394658. The raw proteomics data can be found in the iProX repository, accession number IPX0014917001.
